# Treatment of pustular psoriasis with anakinra: a statistical analysis plan for stage 1 of an adaptive two-staged randomised placebo-controlled trial

**DOI:** 10.1186/s13063-018-2914-y

**Published:** 2018-10-03

**Authors:** Suzie Cro, Catherine Smith, Rosemary Wilson, Victoria Cornelius

**Affiliations:** 1 Imperial Clinical Trials Unit, School of Public Health, Imperial College London, Stadium House, 68 Wood Lane, London, W12 7RH UK; 2 St John’s Institute of Dermatology, Guy’s and St Thomas’ NHS Foundation Trust and Kings College London, 9th Floor Tower Wing, Guy’s Hospital, London, SE1 9RT UK

**Keywords:** Psoriasis, Palmoplantar pustulosis, Randomised controlled trial, Anakinra, Adaptive trial, Statistical analysis plan

## Abstract

**Background:**

Palmoplantar pustulosis (PPP) is a rare, chronic inflammatory skin disease. It is known to affect quality of life at a level comparable to that from major medical and psychiatric illness, yet current treatment options are remarkably limited. Recent evidence however suggests that interleukin-1 (IL-1) blockade with anakinra will deliver therapeutic benefit in PPP.

**Methods:**

Anakinra for Pustular psoriasis: Response in a Controlled Trial (APRICOT) is a two-staged, adaptive, double-blind, randomised placebo-controlled trial which aims to test the hypothesis that IL-1 blockade with anakinra will deliver therapeutic benefit in PPP. During stage 1 a total of 24 patients will be randomised (1:1) to receive either placebo or anakinra. The two candidate primary outcomes are fresh pustule count (across palms and soles) and the Palmoplantar Pustulosis Area and Severity Index (PPPASI) score, recorded at baseline and at weeks 1, 4 and 8. Analysis at the end of stage 1 will compare treatment arms to ensure sufficient efficacy and safety in order to progress to stage 2. The primary outcome for stage 2 will also be identified following an assessment of the reliability and discriminative ability of fresh pustule count and PPPASI. The trial is powered to detect efficacy and will recruit an additional 40 patients in stage 2 (*n* = 64 in total). Analysis will follow the intention-to-treat principle and analyse patients as randomised.

**Discussion:**

This manuscript describes the important features of the small population trial design for APRICOT and the pre-specified statistical analysis plan for stage 1. The statistical analysis plan has been developed prior to data extraction and in compliance with international guidelines. It will increase the transparency of the data analysis for the APRICOT trial. The findings of the trial will help to clarify the role of anakinra in the treatment of PPP.

**Trial registration:**

ISCRTN, ISCRTN13127147. Registered on 1 August 2016. EudraCT Number 2015-003600-23. Registered on 1 April 2016.

## Background

Psoriasis is a chronic inflammatory skin disease with an estimated UK prevalence of 2%. Palmoplantar pustulosis (PPP) is a rare pustular form of the disease which affects the hands and feet and is characterised by painful, intensely inflamed, red skin studded by sheets of monomorphic, sterile, neutrophilic pustules [1, 2]. It is known to affect quality of life at a level comparable to that from major medical and psychiatric illness, yet treatment options for PPP are profoundly limited [3]. With the exception of one small, underpowered randomised controlled trial (RCT) in PPP involving ustekinumab (*n* = 33) [4], no relevant interventional trials have been performed since 2001 (highlighted in the National Institute for Health and Care Excellence (NICE) guidelines on psoriasis) [5]. Recent evidence however suggests a key pathogenic role for interleukin-1 (IL-1), a cytokine that is known to sustain the inflammatory responses initiated by skin keratinocytes [6–8]. Thus, it is hypothesised that IL-1 blockade will deliver therapeutic benefit in PPP.

Anakinra for Pustular psoriasis: Response in a Controlled Trial (APRICOT) is a two-staged, adaptive, double-blind, randomised placebo-controlled trial which aims to test the hypothesis that IL-1 blockade with anakinra will deliver therapeutic benefit in PPP. As proof-of-concept data and safety information is limited in this rare disease (due to the small population), a two-staged design has been adopted. An analysis at the end of stage 1 will compare treatment arms to ensure sufficient efficacy and safety in order to progress to the larger stage 2 study. Since there are no validated outcomes to measure disease change for pustular psoriasis, the primary outcome for stage 2 will be chosen from two candidates following an assessment of reliability and discriminative ability at the end of stage 1. The trial is powered to detect efficacy. Fuller details on the rationale and proof-of-concept data are given in the study protocol [9]. This paper describes important features of the innovative small population trial design and the statistical principles and methods which will be applied to analyse the data at the end of stage 1.

## Methods and design

APRICOT is a small population, randomised, double-blind, placebo-controlled trial of the IL-1 blockade anakinra in PPP with two stages and an adaptive element. The interim analysis for stage 1 will occur after 24 participants have been recruited and completed 8 weeks of follow-up. A decision to embark on stage 2, involving a further 40 participants, will be made using pre-defined STOP/GO efficacy criteria. The primary outcome for stage 2 will also be chosen from two potential candidates (described below) which will be assessed for reliability and discriminative ability at the end of stage 1.

### Primary objective

The primary objective of the APRICOT trial is to determine the efficacy of anakinra in the treatment of adults with PPP compared to placebo. The two candidate primary outcomes are fresh pustule count over palms and soles and the Palmoplantar Pustulosis Area and Severity Index (PPPASI) [10]. The primary endpoint will be change in disease activity over 8 weeks, adjusted for baseline, measured using fresh pustule count over palms and soles, the default primary outcome, unless the PPPASI is assessed to be a more reliable and appropriate measurement. The secondary objectives of APRICOT are detailed in the trial protocol [9].

### Randomisation

Participants with a diagnosis of PPP will be randomised using blocked randomisation stratified by centre and a 1:1 ratio to receive treatment or placebo for 8 weeks. Stage 1 will include approximately four to eight centres (National Health Service (NHS) clinics). Participants will be randomised using an online randomisation system by the King’s Clinical Trials Unit to ensure allocation concealment.

### Intervention and follow-up

Every randomised participant will be required to self-administer anakinra or matched placebo subcutaneous injections daily for 8 weeks. Scheduled visits for follow-up occur at weeks 1, 4, 8, 12 and 20. The primary endpoint is week 8. Follow-up at weeks 12 and 20 will be used to assess disease relapse off study treatment and adverse events.

### Blinding

Participants, treating physicians, research nurses and independent outcome assessors will be unaware of treatment assignment. In trials of anakinra given for other indications, injection site reactions have been reported to occur in 71% of patients compared to 28% in placebo-treated patients [11]. To prevent inadvertent unblinding, the independent outcome assessor will only have sight of the hands and feet, not where the injection is given. Therefore, the assessor will not be able to observe injection site reactions. To achieve this, the patient will be invited into the clinical room by another member of the research team and asked to remain fully dressed apart from the feet and hands. The blinded assessor will then be brought into the room to do the assessment and will not be able to speak to the patient. The independent blinded assessor may optionally wear headphones to avoid inadvertently hearing any discussion between the patient and clinician/other members of the research team to maintain the blind. The analysis of stage 1 will be conducted subgroup blind (i.e. masked as group A versus B) following a pre-specified analysis plan which has been developed prior to any data extraction and is described below. The statistician who conducts the stage 1 analysis will be unblinded on completion of stage 1 analysis. A second statistician will remain subgroup blind throughout the trial and conduct the final analysis subgroup blind. A detailed statistical analysis plan for APRICOT stage 1 will be finalised once the primary outcome for stage 2 has been confirmed.

### Stage 1

#### Stage 1 outcomes

A total of 24 participants with PPP will be randomised in stage 1. The stage 1 outcomes are:

1. Fresh pustule count over palms and soles: to be included in the count, pustules must be macroscopically visible, white/yellow in colour with no brown colour and present on the glabrous skin of the palms and/or soles.
2. PPPASI score: a composite continuous measure encompassing area, pustules, redness and scaling which ranges from 0 (no PPP) to 72 (most severe PPP) [10].

Outcome assessments of fresh pustule count and PPPASI will be carried out at each study site by an independent assessor blind to study treatment at baseline and at weeks 1, 4, 8 and 12. During stage 1 of the trial a second assessor blind to treatment at each site will also use the PPPASI to enable inter-rater reliability to be assessed. Fresh pustule count will also be assessed by a central, blinded assessor using photography (pre-specified views of palms and soles at baseline (visit 1), week 1 (visit 2) and week 8 (visit 3) of treatment). A central comparison is being made to assess whether all sites are counting consistently. PPPASI is a quantitative rating score for measuring the severity of psoriatic lesions based on area coverage, erythema (redness), induration (thickness) and desquamation (scaling). It would not be possible to assess all components via photography. Therefore two on-site clinical raters will take measures of PPPASI to assess the reliability of this potential primary outcome.

**Fig. 1:**
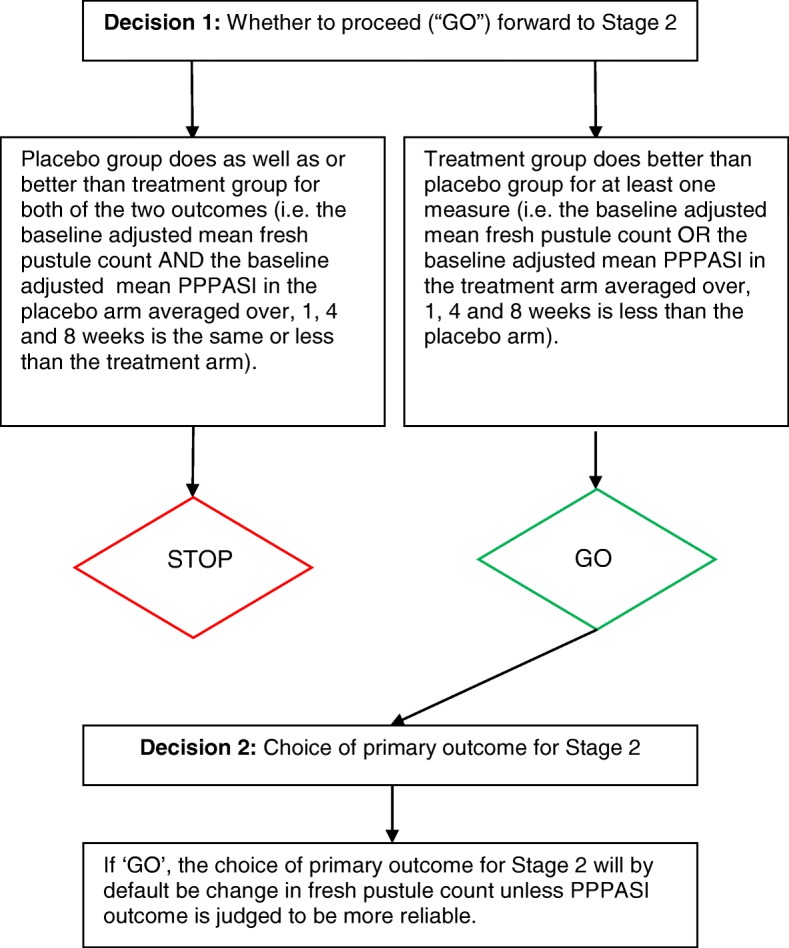
STOP/GO decision criteria to be employed following the completion of 8 week follow-up by *n* = 24 randomised patients

The mean value of the fresh pustule count (assessed at ‘site’) and PPPASI score across follow-up visits (averaged over 1, 4 and 8 weeks for each patient), adjusted for baseline, will be used to inform decision 1 on efficacy (see Fig. 1). The distribution for each outcome across follow-up visits, the agreement between the blinded site assessments of PPPASI and the agreement between the blinded central ‘photographic’ assessment and blinded ‘site’ assessment of the pustule count will be used to inform decision 2 on primary outcome selection for stage 2. The PalmoPlantar Pustulosis Investigators Global Assessment (PPP-IGA) is used to assess eligibility for participant inclusion in APRICOT. The PPP-IGA is a categorical outcome measure with 5 potential levels of: clear/almost clear/mild/moderate/severe. Participants are required to have at least moderate disease on the PPP-IGA. During Stage 1 of the trial a second assessor blind to treatment at each site will also assess the PPP-IGA to establish the reliability of this measure. PPP-IGA will not be considered as a potential primary outcome candidate since continuous outcomes (PPPASI or fresh pustule count) are typically more powerful and offer improved discrimination between treatment groups.

#### Stage 1 STOP/GO criteria

Pre-specified STOP/GO criteria, described in Fig. 1, establish the conditions for progression from stage 1 to stage 2 of the trial (decision 1) and selection of the primary outcome for stage 2 (decision 2). Progression will be determined based on the ordering of the observed mean outcome values in both treatment arms. The trial will qualify for progression to stage 2 to complete the powered efficacy trial provided the treatment group does better than the placebo group for at least one of the candidate primary outcome measures (PPPASI or fresh pustule count).

Assuming the GO criteria are achieved, the Independent Data Monitoring Committee (IDMC) will then review the accumulated safety data from stage 1 and any new drug safety data available through the drug manufacturer Swedish Orphan Biovitrum (SOBI) or other sources to confirm sufficient safety. The IDMC will be responsible for making a recommendation to the Trial Steering Committee (TSC) and the funder, the Efficacy and Mechanism Evaluation (EME) Programme, as to whether the trial should continue to stage 2 and the choice of primary outcome. To maintain blinding, the unblinded efficacy data will only be reviewed by the IDMC, with contributions from the unblinded statistician and funder representation (EME).

### Sample size

The sample size has been calculated using a standardised effect size, as there are two potential primary outcomes. A large effect size of 0.9 standard deviation (SD) has been assumed, with consideration to the cost of the drug and requirement for daily self-administered subcutaneous injections. Large effect sizes have also been reported with oral retinoids [3, 12], a recommended systemic intervention for pustular psoriasis. A sample size of 27 per arm would be required to detect a difference of 0.9 SD with a power of 90% and a 5% significance level. We aim to recruit 32 participants per arm (*N* = 64 in total) to allow for a conservative 15% withdrawal rate. RCTs involving placebo arms [3, 10] have observed a withdrawal rate of less than 5%.

The sample size for stage 1 is based on correct ordering of group means. Stage 1 does not involve statistical tests. We want a high probability of continuing (‘GO’), assuming a conservative true difference in means between the groups of 0.5 SD in favour of the treatment group. Larger differences have been reported. With 20 patients, assuming a real difference of 0.5 SD, the probability that the baseline-adjusted means for treatment arms will be correctly ordered (i.e. treatment > placebo) is 0.85 assuming a distribution of the difference of the two means is normal with mean 0 and variance 2/*n* [13]. If two outcomes are assessed, each with an expected difference of 0.5 SD, then the overall probability of failing to GO is (1 – 0.85)^2^ = 0.0225, i.e. less than 3 in 100. There is thus a minimal chance of failing to continue if the treatment really is beneficial. Based on these rules, if there is no treatment benefit, the probability of not progressing to the next stage is 0.25. Whilst this is low, the balance of errors has been selected to allow optimal identification of treatment benefit and, if there is no treatment effect, can at most be 0.5 under this design. To account for randomisation imbalances, stage 1 will occur after 24 participants have been randomised and followed up for at least 8 weeks. Recruitment will be paused after the 24th participant is recruited to allow for 8 weeks follow-up, analysis and review of the data from stage 1.

### General statistical principles

Stage 1 analysis will be performed after 24 participants have been randomised and have completed 8 weeks of follow-up. No formal statistical hypothesis testing will be performed at this stage. Analysis will be carried out subgroup blind and will follow the intention-to-treat (ITT) principle. That is, all randomised patients with baseline and at least one recorded outcome over 8 weeks will be analysed in the treatment arms to which they were allocated regardless of treatment subsequently received. No imputation for missing data will be performed for the stage 1 analysis. The proportion of missing data for each outcome will be reported. Confidence intervals (CIs) will be two-sided and at the 95% level. All data will be analysed using Stata/IC (StataCorp, College Station, TX, USA) version 14 or above.

### Statistical analysis plan

#### Recruitment

A Consolidated Standards of Reporting Trials (CONSORT) flow chart [14] will be constructed. This will include the number of eligible patients, number of patients agreeing to enter the trial, number of patients withdrawing and lost to follow-up, the number continuing through the trial and the number included in the stage 1 analysis.

#### Baseline comparability of randomised groups

The following baseline characteristics will be tabulated by treatment arm: age, gender, ethnicity, smoking status, PPPASI, fresh pustule count (palms and soles), fresh pustule count (palms), fresh pustule count (soles), total pustule count (palms and soles), PPP-IGA, patient global assessment, Dermatology Life Quality Index (DLQI) and generalised plaque psoriasis (PASI). Continuous variables will be reported as mean (SD) and median (interquartile range, IQR). Categorical variables will be presented using frequencies and proportions (as a percentage). These summaries will be based on observations only, and the number of missing observations will be reported.

#### Adherence to allocated treatment

The number withdrawing from the treatment schedule will be reported by treatment arm and visit along with reasons for withdrawal. Adherence to the 8-week, daily, self-injection schedule is collected by responses to daily text messages (yes or no to indicate daily dose received or not received). Patients will also be asked for a record of their daily injections at each visit. Self-reported adherence to treatment group, collected via text and at visits, will be reported by treatment arm and visit. The adherence to the planned visit windows will be summarised by treatment arm and visit. The use of a potent corticosteroid as a ‘rescue’ medication will also be reported by treatment group.

#### Loss to follow-up and missing data

The number lost to follow-up will be tabulated by treatment arm and visit. The proportions of participants missing fresh pustule count and PPPASI values will be summarised in each arm and at each time point at which the measurement is planned.

#### Adverse event reporting

Adverse events (AEs) will be tabulated separately by type (adverse event, adverse reaction, unexpected adverse reaction, serious adverse event, serious adverse reaction or unexpected serious adverse reaction) and by treatment arm. AEs will be tabulated by treatment arm for both the number of events and the number of participants with events if participants suffer repeated events. AEs will be recorded using terms of the local clinical investigators’ choosing and will be recoded centrally using the Medical Dictionary for Regulatory Activities (MedDRA) for reporting at the ‘Preferred Term’ level.

#### Analysis for decision 1

Primary outcome assessments of fresh pustule count (involving palms and soles) and PPPASI will be carried out by an independent assessor blind to the study treatment at each site. The mean value of each outcome across follow-up visits (averaged over 1, 4 and 8 weeks for each patient) and at each follow-up visit will be summarised to inform decision 1 (see Fig. 1). Baseline-adjusted treatment group differences with 95% CIs will also be presented and calculated using linear regression models. An additional adjustment for centre will be made if there are an adequate number of recruits per centre per treatment arm (e.g. if there are > 5 recruits per centre and/or including centre does not result in unstable model estimates). These summaries will be based on observations only. If the baseline-adjusted mean fresh pustule count OR the baseline-adjusted mean PPPASI in the treatment arm over 1, 4 and 8 weeks is less than that in the placebo arm, the trial will continue to stage 2. No formal statistical testing will be conducted at this point, as the sample size has been determined to detect the correct ordering of the means and not to test statistical significance.

#### Analysis for decision 2

We will look at how reliable fresh pustule count and PPPASI are at the end of stage 1. The distributions of the outcome assessments of fresh pustule count and PPPASI will be assessed using histograms by treatment group at each assessment point. The mean value of each outcome will also be plotted across time by intervention arm in a scatter plot alongside individual patient outcomes.

The standardised mean difference (SMD) will be reported for each outcome and assessment point (difference of the group means divided by the pooled SD of the group means). The SMD is unitless and can be used to compare the discriminatory ability across the various measures. The higher the value of the SMD, the greater the discriminatory ability. Accompanying 95% CIs, constructed using the noncentral *t* distribution [15], will be presented alongside the SMD.

The agreement between the ‘site’ assessors and the central ‘photographic’ assessment will be assessed using the method of Bland and Altman [16] allowing for the multiple observations. That is, we will visually inspect a plot of the difference between the two assessors for each assessment measuring the same quantity, i.e. for the same patient and same time, against their mean and present 95% limits of agreement calculated as *d* +/− 1.96*s*, where *d* denotes the mean difference and *s* denotes the standard deviation of the differences. The limits of agreement represent the region in which 95% of differences will lie (assuming normality of differences). Each patient has photographs taken at baseline, week 1 and week 8. Therefore, we will have a maximum of 72 (3 × 24) pairs of assessments to compare. We will also plot differences separately by site and assess agreement within each site where data is sufficient. This analysis assumes the repeated differences for each patient are independent over time, i.e. that there is no autocorrelation. As recommended by Bland and Altman [16], we will perform a visual check on the validity of this assumption in a plot of the observed differences against time, differentiated by patient. If the assumption is violated, we will subsequently assess agreement separately by time.

A systematic difference in fresh pustule count between the ‘site’ and ‘photographic’ assessment may be observed due the potential for pustules that extend immediately beyond but are contiguous with the palms and soles to be included in the ‘site’ assessment but not the ‘photographic’ assessment. The individual variability of the measures (precision) will be of more importance with respect to achieving good discrimination than the absolute agreement in count. As a result, we will calculate a measure of consistency comparing the ‘site’ and ‘photographic’ assessment using the intraclass correlation coefficient (ICC). This will be calculated using a mixed-effects analysis of variance (ANOVA) with a random intercept for patient and rater (‘site’ or ‘photographic’). The model will include a fixed effect for time to allow for a different mean response over patient and rater by time. The variability of rater will not be included in the denominator of the ICC, as this provides an estimate for consistency [17].

For the fresh pustule count for participant *i* assessed by rater *j* at time *k*, denoted as *y*_*ijk*_, the model to be fitted will therefore be:

$$ {y}_{ij k}={\beta}_0+{\beta}_1{x}_1+{\beta}_8{x}_8+{c}_i+{r}_{ij}+{e}_{ij k}, $$

for

*i* = 1 to 24 patients,

*j* = 1 to 2 raters (1 = ‘site’, 2 = ‘photographic’),

*k* = 0 (baseline), 1 or 8 weeks,

*x*_*k*_ is a dummy variable for time point *k* weeks (=0 or 1) for *k* = 1 or 8,

*β*_0_ is a regression coefficient representing the average outcome at baseline,

*β*_*k*_ is a regression coefficient representing the average difference in outcome at time point *k*, relative to baseline for time *k* = 1, 8 weeks,

$$ {c}_i\sim N\left(0,{\sigma}_c^2\right), $$

$$ {r}_{ij}\sim N\left(0,{\sigma}_r^2\right), $$

$$ {e}_{ijk}\sim N\left(0,{\sigma}_e^2\right). $$

${\sigma}_c^2$is the variance of the measurements across subjects, ${\sigma}_r^2$is the variance of the measurements across the two methods for the same patient and ${\sigma}_e^2$ is the unexplained residual variance of the measurements for the same patient and method. The ICC which represents the extent to which there is a consistent agreement among raters will be estimated as:

$$ ICC=\frac{\sigma_c^2}{\sigma_c^2+{\sigma}_e^2}. $$

If the unexplained measurement variability is small in comparison to the true differences between the patients, the ICC will be closer to 1. This analysis assumes the repeated patient outcomes are independent over time, i.e. that there is no autocorrelation. We will perform a visual check on the validity of this assumption in a plot of outcomes against time, differentiated by patient. If the assumption is violated, we will also fit a separate model for each time point k as:

$$ {y}_{ij}={\beta}_0+{c}_i+{r}_{ij}+{e}_{ij} $$

and calculate and present the ICC by time. The agreement between the first and second assessor PPPASI measures, recorded at site, will be assessed using the same methods outlined above (Bland and Altman and ICC from mixed-effects ANOVA). The distribution for each outcome across follow-up visits, the agreement between the two site assessor measures of PPPASI and the agreement between the blinded central outcome assessor and blinded site outcome assessor for pustule count will be used to inform decision 2.

The agreement between the first and second assessor assessments of PPP-IGA will be assessed using Cohen’s weighted kappa statistic, κ, with a linear weighting scheme so that the degree of disagreement between pairs of readings is proportional to the number of grades apart. Weighted kappa values can vary from − 1 (complete disagreement) through 0 (chance agreement) to + 1 (complete agreement). Intermediate kappa values will be interpreted using the criteria described by Landis and Koch [18], where 0 < κ ≤ 0.20 = slight agreement, 0.20 < κ ≤ 0.40 = fair agreement, 0.40 < κ ≤ 0.60 = moderate agreement, 0.60 < κ ≤ 0.80 = substantial agreement and 0.80 < κ < 1 = almost perfect agreement.

## Discussion

In small populations, such as PPP, innovative trial designs are necessary to ensure treatment progression is achieved. An additional challenge in this population was the lack of validated measures of disease activity. APRICOT has been designed to meet these needs.

In this article we have described the analysis for stage 1 of APRICOT, which will facilitate decision-making for progression to stage 2 and the most suitable as well as reliable primary outcome measure for stage 2. This pre-specified statistical analysis plan will increase the transparency of the data, analysis and reporting.

## Abbreviations

AE: Adverse event
ANOVA: Analysis of variance
APRICOT: Anakinra for Pustular psorias: Response in a Controlled Trial
CI: Confidence interval
CONSORT: Consolidated Standards of Reporting Trials
DLQI: Dermatology Life Quality Index
EME: Efficacy and Mechanism Evaluation (Programme)
ICC: Intraclass correlation coefficient
IDMC: Independent Data Monitoring Committee
IQR: Interquartile range
ITT: Intention-to-treat
MedDRA: Medical Dictionary for Regulatory Activities
PPP: Palmoplantar pustulosis
PPPASI: Palmoplantar Pustulosis Area and Severity Index
PPP-IGA: Palmoplantar Pustulosis Investigators Global Assessment
SD: Standard deviation
SMD: Standardised mean difference
SOBI: Swedish Orphan Biovitrum
